# Big data-driven public health policy making: Potential for the healthcare industry

**DOI:** 10.1016/j.heliyon.2023.e19681

**Published:** 2023-08-31

**Authors:** Kang Chao, Md Nazirul Islam Sarker, Isahaque Ali, R.B. Radin Firdaus, Azlinda Azman, Maslina Mohammed Shaed

**Affiliations:** a School of Economics and Management, Neijiang Normal University, Neijiang, 641199, China; b School of Social Sciences, Universiti Sains Malaysia, USM, Pinang, 11800, Malaysia; c Department of Development Studies, Daffodil International University, Dhaka, 1216, Bangladesh

**Keywords:** Big data analytics, Data-driven policy, Policy process, Public health policies, Health communication

## Abstract

The use of healthcare data analytics is anticipated to play a significant role in future public health policy formulation. Therefore, this study examines how big data analytics (BDA) may be methodically incorporated into various phases of the health policy cycle for fact-based and precise health policy decision-making. So, this study explores the potential of BDA for accurate and rapid policy-making processes in the healthcare industry. A systematic review of literature spanning 22 years (from January 2001 to January 2023) has been conducted using the PRISMA approach to develop a conceptual framework. The study introduces the emerging topic of BDA in healthcare policy, goes over the advantages, presents a framework, advances instances from the literature, reveals difficulties and provides recommendations. This study argues that BDA has the ability to transform the conventional policy-making process into data-driven process, which helps to make accurate health policy decision. In addition, this study contends that BDA is applicable to the different stages of health policy cycle, namely policy identification, agenda setting as well as policy formulation, implementation and evaluation. Currently, descriptive, predictive and prescriptive analytics are used for public health policy decisions on data obtained from several common health-related big data sources like electronic health reports, public health records, patient and clinical data, and government and social networking sites. To effectively utilize all of the data, it is necessary to overcome the computational, algorithmic and technological obstacles that define today's extremely heterogeneous data landscape, as well as a variety of legal, normative, governance and policy limitations. Big data can only fulfill its full potential if data are made available and shared. This enables public health institutions and policymakers to evaluate the impact and risk of policy changes at the population level.

## 1 Introduction

The possibilities and advantages of big data in the healthcare sector are apparent, and its usage is expanding globally. Big data is mostly used to describe data sets that are too big or complicated for conventional data-processing application software. Greater statistical power is provided by data with more fields or greater complexity, but this statistical power may also increase the incidence of erroneous discoveries. Large-volume datasets may be utilized via big data analytics (BDA) [1] to enhance diagnosis, guide preventative medical procedures and lessen the negative effects of medication and other forms of therapy. Across a range of healthcare contexts and areas, big data is having an impact. The term “BDA” encompasses the views of big data and analytics. Generally four aspects known as 4 V s (volume, variety, veracity, and velocity) were emphasized in the case of healthcare big data [2,3]. However, recent studies indicate that these aspects have expanded to more than ten, comprising variability, value, viscocity, volatility, viability, validity, and others, depending on the particluar context [4,5]. To reiterate, the phrase “big data” describes more than just volume; it also emphasizes the analytical burdens related to a particular mix of data variety and velocity [6]. This research recognises this change and concentrates on the fundamental aspects that are especially pertinent to the formulation of health policy.

There are two types of literature found on BDA and health policy-related issues; one is on the use of BDA for health policy-making decisions [[7], [8], [9], [10], [11]], and another is on how public health policy can advance the use of big data [12,13]. Both are opposite to each other but we focus on the first one due its synergy with the goal of our study. For example, Mählmann et al. [7] and Vassiliou et al. [8] contend that big data can only be beneficial if the data are made accessible. Anisetti et al. [9] concentrate on using big data to evaluate public health policies, particularly for reducing the cost of clinical trials and facilitating evidence-based decisions. On the other hand, Heitmueller et al. [12] and Vydra and Klievink [13] emphasize the use of public policies to govern Big Data Analytics (BDA) within the healthcare industry.

Large and sophisticated electronic data sets that cannot be managed by conventional hardware or software are referred to as “big data” in the healthcare industry. As contemporary technology is used more often in healthcare systems, huge amounts of big data are being produced [14,15]. Although the amount of health data has always been substantial, recent technology advancements have increased its diversity, pace and volume. The technical advancements surrounding big data, which have enabled the healthcare industry to increase the quality of its data and add value, are even more exciting [16]. Healthcare institutions may use big data analytics to find patterns and trends in data to enhance patient care, save lives and cut costs. With the aid of big data and analytics, those managing healthcare systems can focus on enhancing results (both health-related and monetary), population health analysis, and expediting clinical decision-making. For example, a platform called CrowdHEALTH was proposed by Kyriazis et al. [17], and it was built on the principles of Data & Structures, Health Analytics, and Policies. Holistic Health Records (HHRs), a new paradigm for describing health status utilizing big data management techniques, were developed. A growing body of literature covers the use of big data for health related decision-making [18]. The research, however, does not fully explore how healthcare stakeholders perceive the use of big data in healthcare.

Using big data in creating public health policies brings a new age of accuracy, effectiveness, and inclusion that will significantly benefit a wide range of stakeholders [19]. Public health organizations may use large databases to spot epidemiological patterns, predict disease outbreaks, and allocate resources as efficiently as possible, improving public health responsiveness and readiness. For example, Biran et al. [20] proposed PolicyCLOUD platform which facilitates the flexible exploitation and management of policy-relevant dataflows by allowing practitioners to register datasets and define a series of transformations and/or information extraction using registered ingest functions. Using a newly proposed type of electronic health records and their corresponding networks, Mavrogiorgou et al. [21] proposed beHEALTHIER, which provides the capacity to generate health policies out of data of collective understanding through the effective management of healthcare data. Data-driven policies guarantee that health interventions are transparent, timely, and personalised for the general public [22]. This promotes confidence and compliance with public health directives. Healthcare providers may also adjust their strategy to ensure that services match the changing requirements of their communities by using actionable information. From a wider angle, big data-driven public health policies create a more resilient, egalitarian, and health-promoting society where all sectors of society come together to pursue holistic well-being.

The World Health Organization (WHO) defines health policy as “decisions, programs, and activities undertaken to accomplish specified health care goals within a society." Despite the need that policies be founded on scientific evidence, evidence-based decision-making is still the exception rather than the norm in many nations, especially low- and middle-income ones [23]. Internal statistics, reports and opinions of internal staff members are the types of information that are most commonly utilized instead of research evidence, even in high-income nations. However, evidence-based policies require providing large-scale data to produce trustworthy projections for future actions. On the one hand, there are situations when the proper data is lacking. Currently, public health policy-making attempts to create health-promoting and preventive initiatives in order to promote a more proactive approach to healthcare [24]. This is due to several profound societal changes, including the dynamics of population age structure, technological advancements and the burden of chronic diseases. Though possibly unsustainable, total spending tends to increase in the healthcare sector considerably more quickly than in non-health related industries. In addition to being valuable in and of itself, good health is a requirement for economic development; therefore, effective health expenditure may boost economic growth. On the other hand, every choice, arrangement or action performed to advance certain healthcare objectives inside society is referred to as public health policy-making. In order to safeguard health as a value, it attempts to define visions for the future, which in turn helps develop objectives, points of reference and priorities.

A public authority must possess data science-related skills and expertise to fully exploit big data in the public sector [25]. Computer modeling, statistics, data management, data exploration, algorithmic machine learning, data product formatting and other topics are included in this emerging discipline. Programming for computers is also a part of it. Additionally, the public sector requires a different strategy than the private sector, necessitating the customization of strategies according to the general public's aims, objectives and policies. Despite the possible labor requirements, big data has enormous promise for public administration and the common good [26].

There is a shortage of comprehensive, publicly available research on big data theory and applications in health administration sector. However, tangible evidence of significant results exists. These instances make it possible to analyze the use of big data, draw general conclusions and pinpoint prospective public health sector use of BDA-driven policy making [27]. Recently, many studies have been conducted in clinical settings and public health sectors by applying the concept of “big data” and “data analytics” [19,20]. Furthermore, government organizations use BDA more often to solve issues like pandemics and sustainability [30]. Nevertheless, there are obvious research gaps, and there is not a clear vision of how big data may be used in making public health policy.

Governments may better understand the behavior of their citizens and improve public services by using new approaches for big data analysis [31]. Big data may be used in a variety of ways to improve public health sector performance, according to the most recent studies on the subject [26,32,33]. These include increasing government efficacy [34], openness [35] and efficiency [36], as well as making evidence-based policy decisions [37] and providing better services based on an improved understanding of the specific needs of residents [38]. The use of big data, as observed by academics, holds great potential for several policy areas, including health care [39], the economy [40], the environment [41] and transportation [42]. Although big data solutions are promoted to solve social issues, numerous questions concerning the breadth and potential of big data and how, where and when they are likely to be beneficial remain unresolved [34].

Several previous studies used evidence-based methods and verified their results, such as predictive models, in big data analytics (BDA) for developing public health policy. Patients with high risks and high costs were identified by Bates et al. [43] using evidence-based procedures. Early detection of these individuals may decrease unnecessary hospitalizations and treatments, improving the cost-effectiveness of health efforts. They used large amounts of data from electronic health records and verified their prediction model for locating such individuals, which has significant ramifications for healthcare policy. According to Jiang et al. [44], using artificial intelligence (AI) in healthcare data may aid in determining patient requirements, forecast illness development, and directing treatment regimens. The prediction models were verified by using information from electronic health records. Murdoch et al. [45] emphasized using evidence-based methods for deciphering huge, complex datasets and offered examples of validated prediction models in the medical field. The development of effective and efficient public health policies may benefit from using BDA and its auxiliary tools, such as predictive models. Raghupathi and Raghupathi [46] explained how to use BDA to concentrate on characteristics that are most likely to lead to better public health outcomes. They identified essential health determinants by analysis of large healthcare databases, which enables the development of efficient health programs that target these important areas, maximizing the potential for improving health outcomes. Cost-effectiveness, risk free, and outcome-oriented public health policies' can be promoted by using these evidence-based practices.

Furthermore, the public health sector is only just beginning to use big data for health policy decision-making. Most big data efforts are still in the early phases of development or are only in the planning stages. Effective public health policy choices can be aided by good data utilization. Many studies are being done on the application of big data, particularly in respect to policy cycle, to assist evidence-based policymaking in various sectors. In line with the theoretical model of the policy process, evidence-based public health policy makes sense. The use of big data to assist an informed choice at different phases of the public health policymaking process has not been thoroughly studied, and only a small number of academics have looked at the possible uses of big data in different phases of the policymaking process in different sectors [26,28,36,[47], [48], [49]]. To fill the previously noted research gap, the use of big data in various phases of the public health policy process is studied in this study. Consequently, the overarching research question that motivates and guides our effort are: What are the potentials of BDA in making public health policy? And What are the barriers to using BDA in public policymaking execution? Therefore, this study explores the potential of BDA for promoting accurate and rapid policymaking processes in the healthcare industry. The findings will aid in understanding the data sources of public policy, the use of big data in policy analysis stages, the indicators for effective public policy and the problems associated with using big data in public policy areas. These insights can assist administrators, stakeholders, policymakers and people in formulating sound public policy.

## 2 Materials and methods

**Fig. 1:**
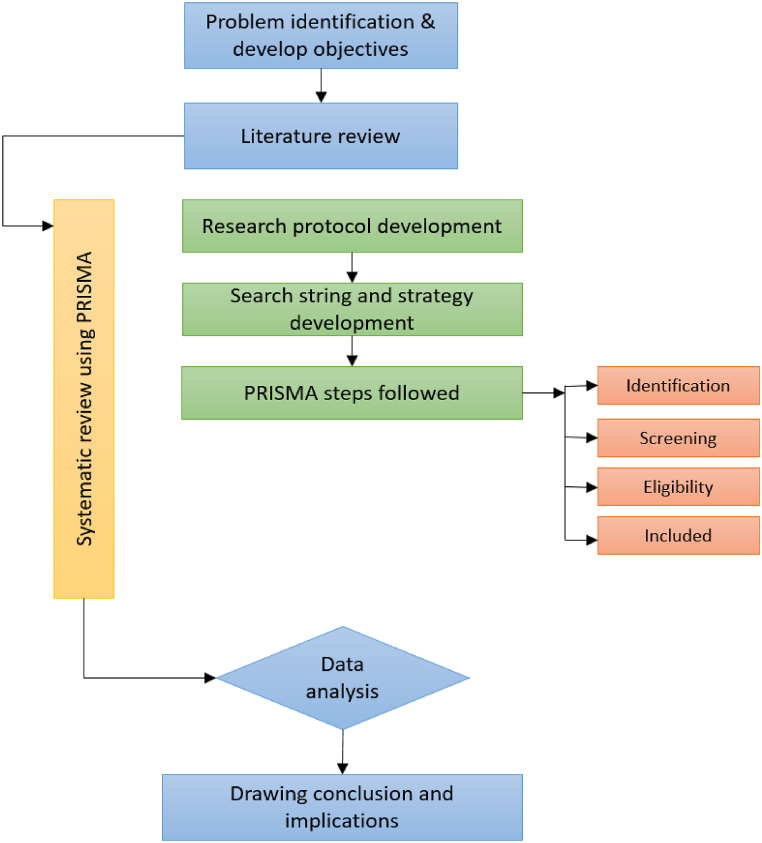
Flow diagram of the research process.

Data were collected utilizing a systematic review methodology that used credible sources. According to Saunders and Lewis [50], a systematic literature review begins with defining relevant keywords to search and retrieve information from databases. Conferring to Tranfield et al. [51], a literature review seeks to uncover knowledge gaps and limitations. A literature review also categorizes and evaluates previous research based on significant concerns and recommendations for future research. The current inquiry adhered to the PRISMA (preferred reporting items for systematic reviews and meta-analyses) technique of reporting components, including the creation of a research protocol. The PRISMA method is well-known for conducting systematic reviews and meta-analyses based on evidence. It comprises a 27-item checklist and its four primary processes: identification, screening, eligibility and inclusion. Assessment of strong and weak areas, visualization of document identification quality, and replication of its structure and formatting are the major features of the PRISMA methodology. Fig. 1 shows the steps of the research process comprising all essential stages.

The following parts explain the current study's research design, database selection rationales, publication criteria, timeframe, search strategy, search fields and inclusion and exclusion criteria.

### 2.1 Research design

This study adopted a systematic review methodology, which comprises developing a research protocol and identifying the most relevant material. According to Fink [52], a systematic literature review is methodical, particular, extensive and reproducible for locating, assessing and synthesizing the existing body of knowledge. The PRISMA framework represents the basis for the data collection suggestions [53] and is commonly adopted for conducting systematic literature reviews. Identification, screening, eligibility, and inclusion are the four primary phases of the PRISMA methodology. PRISMA checklists were utilized to complete each of these four steps.

### 2.2 Eligibility criteria

The article's intended keywords, language and publication date were examined as inclusion criteria. Only English-language, peer-reviewed journal articles were considered for the investigation. This analysis covers the period from January 1, 2001 to January 20, 2023.

### 2.3 Research protocol

The established research protocol ensured the inquiry is scientific (Table 1). A detailed review of the existing literature published during the preceding 22 years (January 1, 2001 to January 20, 2023) to substantiate claims for applying big data in public policy and administration practices.

**Table 1:** Research protocol details.

Phases	Details
Selected database	Scopus
Criteria of publication	Peer-reviewed article
Language	English
Period covered	January 1, 2001–January 20, 2023
Keywords for search	Big data, big data analytics, public health policy, health policy analysis, health policymaking, health policy decision
Search Fields	Title, abstract, and keywords
Inclusion criteria	The article should have “big data, big data analytics, public health policy, health policy analysis, health policymaking, health policy decision”.
Exclusion criteria	No full text, repetition and non-English article. Articles without “Big data, big data analytics, public policy, policy analysis, policy making” are also excluded.

### 2.4 Searching strategy

Literature reviews enable the analysis and synthesis of prior research, probing its emphasis to unearth fresh information that can help develop a new paradigm for teaching and research. A thorough systematic review was carried out using the PRISMA method [36]. With these characteristics in mind, our research searched the well-known Scopus database for phrases like “big data, big data analytics, public health policy, health policy analysis and health policymaking.” There are numerous databases, but not all of them are equally valuable. Although it excludes certain published articles owing to its qualitative selection procedure, the Scopus database is frequently utilized in literature reviews because of the excellent quality of the papers and publications that are indexed in it. Moreover, reputable databases such as Web of Science, Engineering Village and Inspec, all contained most of the research articles published in the Scopus database. Consequently, this database was chosen as the information source for the current investigation. The search strings used in this study, conducted in January 2023, are displayed in Table 2.

**Table 2:** Searching string of the study.

Database	Final search string	Retrieval time	Final selection
Scopus	(TITLE-ABS-KEY ((big AND data) AND (health AND policy)) AND TITLE-ABS-KEY (big AND data AND analytics) AND TITLE-ABS-KEY (public AND health AND policy) AND TITLE-ABS-KEY (health AND policy AND decision) AND TITLE-ABS-KEY (health AND policy AND making)) AND (LIMIT-TO (DOCTYPE, “ar”) OR LIMIT-TO (DOCTYPE, “re")) AND (LIMIT-TO (LANGUAGE, “English"))	January 20, 2023	11
From references	During abstract screening	January 20, 2023	8

### 2.5 Criteria for inclusion and exclusion of papers

Two criteria were utilized to choose the publications for this study: (a) Does the work contain big data, BDA, public health policy or policy-making decisions? Do public health policy, policy making, policy process and public health policy decisions have any connections? This study used the chosen research methodology to locate the most pertinent publications.

### 2.6 Data analysis

To improve the rigor of the coding process, MAXQDA software was used to do the qualitative analysis. The software allows users to directly annotate texts with codes, code them and then create syntheses using those codes [54]. Utilizing the tool makes the analysis clearer and more trustworthy. The practical coding technique was carried out by one researcher, who read the five-eigenvector core full-text texts from each cluster and annotated them in accordance with the framework.

The objective of the second researcher was to validate the annotator's conclusions. The researchers communicated with one another to ensure that all of the coding schema's parts were understood. The second researcher reviewed the papers and codings produced by the first researcher to ensure that each schema item, if available, was recognized. Although publications frequently reuse the same information, our strategy was designed to avoid collecting it more than once for each publication. Because of the chosen technique, it was impracticable to employ, say, an inter-coding agreement.

The coding MAXQDA analysis tool was used to automatically create the cross tabulations of the coded documents and the synthesis document detailing the coded text segments [26]. These were used in the interpretation process. MAXQDA provided a synthesis of the coded parts to analyze the results. The two researchers independently familiarized the MAXQDA descriptions. After having evaluated the synthesis independently, the researchers discussed their results, and after concluding their coding results, the authors then synthesized the major themes for further research.

### 2.7 Visualization of connected components

**Fig. 2:**
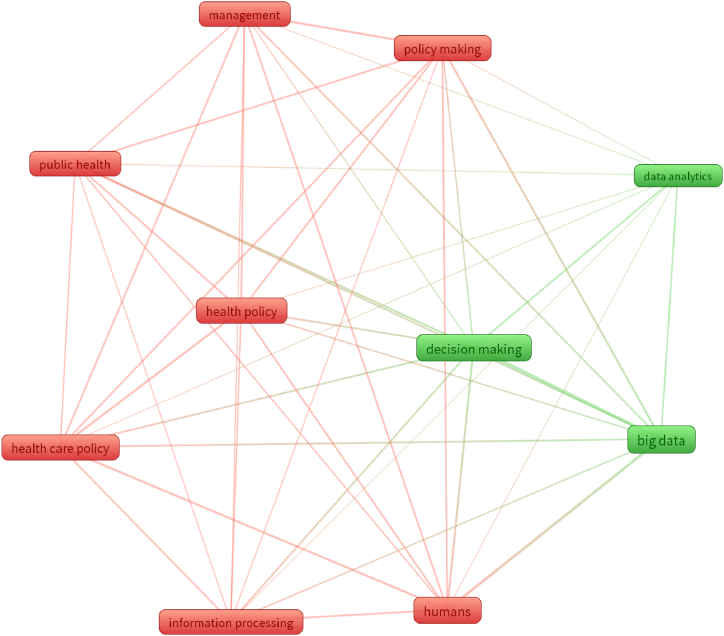
Network visualization of data-driven public health policy.

The word clouds were produced using the VOS viewer application. Bibliographic coupling refers to the sharing of one or more references between two publications. The probability that two items belong to the same cluster increases with the degree of reference overlap between them. The VOS viewer generates a co-occurrence matrix based on similarity evaluations that displays a two-dimensional map of all grouped items. The tighter the interactions are, the stronger they are between the elements in the matrix. The clusters were arranged using an interpretative technique. These interpretations, together with a keyword analysis based on author keywords, were utilized to look into the current hot topics in the scientific literature. The purpose of a word cloud is to provide a concise overview of big data, big data analytics, public health policy, policy analysis, policy making and the connections among these topics. The search approach aimed to understand the relationship between big data, big data analytics, public health policy, health policy analysis and health policy making. To gather preliminary insights on the focal area due to limits, a number of keywords were employed in searches against categories, such as article keywords, title and abstract. Fig. 2 displays the most popular search terms in big data and public policy studies. A phrase appears at least five times in data from publications that have been extracted.

## 3 Results

### 3.1 Identification of documents

**Fig. 3:**
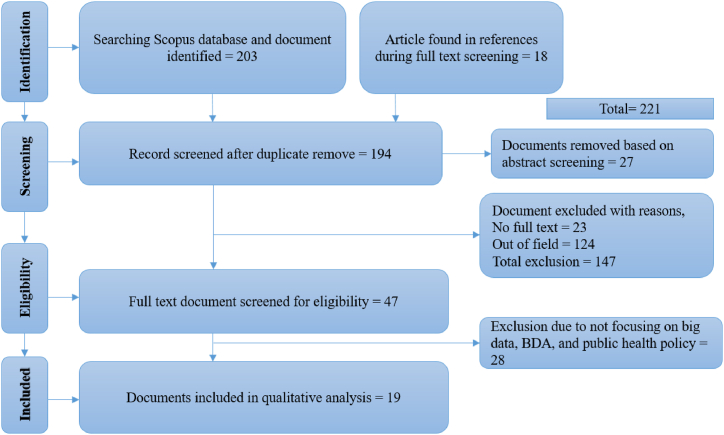
Document identification through PRISMA.

The guidelines from the PRISMA checklists were used to guide this investigation. The PRISMA method consists of four basic steps: identification, screening, eligibility and inclusion. During the identification phase, 203 papers were discovered in the Scopus database, and 18 more were discovered via referrals. The first search produced books, book chapters, journal articles, conference papers and book results. During the screening process, all further papers were excluded with the exception of journal articles. Twenty-seven (27) articles were dropped at the screening stage after the abstracts were examined. At the eligibility stage, 47 papers were picked after disqualifying 147 for not meeting the requirements. For inclusion, 19 most relevant papers were chosen, including real journal articles that might show how big data and data analytics may be used for the formulation and execution of public policy (Fig. 3).

### 3.2 Sources of public policy-related big data

Big data refers to data volumes larger than those that can be handled quickly by commonly available technology, requiring a more specialized and sophisticated analytics approach. In public health policy decision-making, big data analytics (BDA) may be useful for making real-time accurate decision. These methods use enormous data sources to predict what would have happened in certain scenarios. The listed sources include public census data, in-person interviews, crowdsourced information, private sector transactions, social media platforms, website requests from the government, public health sector, tax data, and routine inquiries from various stakeholders to public agencies (Table 3).

**Table 3:** Sources of public health policy-related data.

Major sources	Description	Cited sources
Electronic health record (EHR)	HER is a good source of health-related data.	[5,6,46]
Public health record (PHR)	PHR is a common source of patient data.	[48,56]
Administrative data	Various censuses conducted by the public agency	[51,58,59]
Patient survey	Official face-to-face interviews with public officials	[11,54,61]
Sensing data	Crowdsource data managed by a public agency	[57,63,[64], [65]]
Clinical data	Private sectors like NGOs, banks and development organizations	[28,59,66]
Social networking sites	Various social networking sites, like Facebook, Twitter, LinkedIn, Instagram, WhatsApp and others	[48,61,[68], [69]]
Government website queries	Various queries from the people to a government agency through emails, comments and asking.	[26,39,62]
Insurance claims data	Various insurance data from the insurance company	[64,71]
Medical research and relevant literature	Tax profile of people	[73]
Omics data	Daily queries from people, internal and external users to a public agency.	[49]
Fitness devices	Various fitness devices record user's data.	[57]
Mobile phones	Mobile phones are a common tool for getting general data about its user.	[28,[48], [70]]

### 3.3 Stakeholders of BDA-driven public health policy

The stakeholders involved in applying big data to health policy go beyond healthcare providers, patients, and research institutions to include various entities, and other groups that aren't necessarily directly involved in health research or healthcare services. In order to better understand how new analytical models, data sources, and stakeholders are forming dynamic interactions, it may be helpful to think of the big data in the context of health as an evolving ecosystem (Table 4).

**Table 4:** Public health policy stakeholders.

Major stakeholders	Key indicators	Sources
Individuals and group	Citizens	[5,20]
	Consumers	[74,75]
	Patients	[76,77]
	Civil society	[19,20]
Health services	Health-care providers and institutions	[6]
	Public and private agencies	[[78], [79], [80]]
	Professional institution	[57]
Research and academia	Research institutes and networks	[7]
	Academic institutes and universities	[8]
	Registries	[6]
Health-care industry	Insurances company	[39]
	Pharmaceutical company	[5,6]
	Biotechnology company	[55]
	Health technology company	[33]
	Biobanks	[4,48]
Data and ICT industry	Standards organizations	[[74], [81]]
	ICT businesses	[55]
	Telecommunication	[33]
	Security	[57]
	Analytics marketing	[6]
Government	Public agencies	[[55], [82], [83]]
	Regulators	[[33], [84], [85]]
	Technology agencies	[57]
	International organizations	[6]

### 3.4 Tools of BDA for public health policy

Technology may be used to collect a sizable volume of diverse, high-quality data. BDA entails the modeling, processing, interpretation, analysis, and verification of this data in order to provide valuable information. There are three types of big data analytics: descriptive, predictive and prescriptive [79] (Table 5).

**Table 5:** Usage of BDA for the execution of public policy.

Major analytics	Key venues	Sources
Descriptive analytics	Descriptive analytics compiles historical data to find answers, spot trends in current operations and prepare the data for future analysis. Descriptive analytics is the basis for measuring occurrences, reporting and qualifying massive data for useful insight. By predicting the number of certain patients in a region, this form of analytics may be used to manage population health or pinpoint places where treatment may be improved.	[7]
Predictive analytics	Predictive analytics is used to foresee and comprehend potential future events by examining historical data patterns and trends. It can be used to forecast the weather, identify health services seeking people, and forecast outbreaks and epidemics.	[29,48]
Prescriptive analytics	Prescriptive analytics aid in choosing the optimal course of action. It is the most sophisticated kind of analytics, which goes beyond predicting what will happen in the future since it might help discover the most efficient risk-reduction tactics.	[2,20]

### 3.5 BDA for public health policy formulation

Numerous studies show how big data may be utilized to improve the entire policymaking process. Big data enhances the information intake for decision-making and provides more prompt feedback on policy and its consequences. Big data has immense promise as a tool for informing many phases of the policy analysis process, from issue conceptualization to continuing policy review and even empowering stakeholders and citizens. This part investigates how big data may be utilized in the four stages of the policymaking process in accordance with the literature reviewed. This study has highlighted four key phases in the entire process of public policy formulation where BDA may have a possible function (Table 6).

**Table 6:** Public health policy formulation and BDA.

Major phases	Key indicators	Sources
Problem identification	• Finding a condition that needs fixing and gathering data to show how bad it is. • Finding the important, relevant information to describe the issue.	[7,8] [67]
Agenda setting	• The target audience for this material includes decision-makers as well as other stakeholders. It requires establishing the significance of an issue and its causes. It needs demanding frameworks.	[13]
Policy formulation	• Outlining the mechanisms and regulations that will enable intervention. • Analyzing the effects of current or prospective policies and recording how they will affect health and its determinants (using, for example, tools such as health impact assessments). • Outlining the effects of every choice. Using the data produced by forecasting, describing the future costs and advantages of all strategic options.	[11,73]
Implementation	• Monitoring the consequences of previously passed policies and participating in their implementation. • Producing analyses while using technical proficiency, real-world experience and specialist knowledge, concentrating on the potential use of the knowledge acquired across various contexts.	[13,55] [74]
Policy evaluation	• Developing surveillance systems. • Outlining distinctions between the policy's planned and actual impacts. • Executing complex evaluations.	[82] [11] [74]

### 3.6 Major challenges for governance of BDA for public health policy

Exploiting the potential of these already-existing and quickly-emerging data sources necessitate overcoming many obstacles typical of today's extremely varied data environment as well as various legal, normative governance and policy restrictions. As a result, in addition to technological and infrastructure issues, moral, governmental and social issues need to be resolved [7]. Several challenges have been identified in this study and presented in Table 7.

**Table 7:** Major challenges of data-driven public policy.

Major challenges	Description	Sources
Privacy and security	The security and privacy of its residents are a top priority for the government. The government organization has strict restrictions and instructions on how to use individuals' personal information. The general people should keep believing in government institutions as a secure repository.	[6,64,75]
Data sharing challenges	Data has three basic characteristics: discoverability, usefulness and accessibility. Access to information is essential for building a sustainable economy. While gathering, acquiring, using and maintaining data, the government should adhere to privacy rules. Government organizations rely on big data analytics to make judgments swiftly. For data to useful, it must be perfect, comprehensive and supplied on time.	[28,56]
Technology related challenges	Some years back, big data technologies were unreachable. However, it is now being used more and more in every business. Technology advancements have made it possible to handle, store and analyze vast amounts of data. For data collection, processing, analysis and storage in the public sector, enhanced hardware and software solutions are needed. The challenges and risks must be effectively managed by the government agency to profit from the technology.	[26,56]
Skill-related challenges	Big data management is a relatively new technology and requires a team of qualified personnel that have a deep understanding of several other subjects, including data science. To properly manage data as well as prevent data disaster, government agencies should employ qualified data scientists, who are currently few in the in government organizations.	[28,52]
Challenges with data modeling	There is a shortage of data models describing aspects unique to a subject while meeting data representation requirements. Languages for managing data uncertainty, languages for defining and querying data quality information, and languages for representing and querying data provenance and contextual information need to be improved simultaneously.	[47,60]
Data management issues	It is critical to provide high-quality, affordable, dependable data preservation and access while safeguarding relevant data's privacy, security and intellectual property rights. To develop open-linked data spaces, it is crucial to sustain data search and discovery across various sources and connect data sets from different domains (unstructured or semi-structured). Data and task parallelization is required because massive data sets may be so large that they cannot be handled efficiently by a single computer. As a result, disparate data entry methods, vocabularies and various data formats or labels applied to the same things must be reconciled.	[8,13,60]
Issues with data services and tools	Most scientific fields lack the necessary data analytics tools to support research at all levels. Scientists are, hence, usually less productive than they may be. This demonstrates the urgent need for ICT tools that can “clean,” “analyze” and “visualize” enormous amounts of data, as well as for data tools and policies to guarantee cross-fertilization and collaboration among several disciplines and scientific sectors.	[15,71,72]

## 4 Discussion

**Fig. 4:**
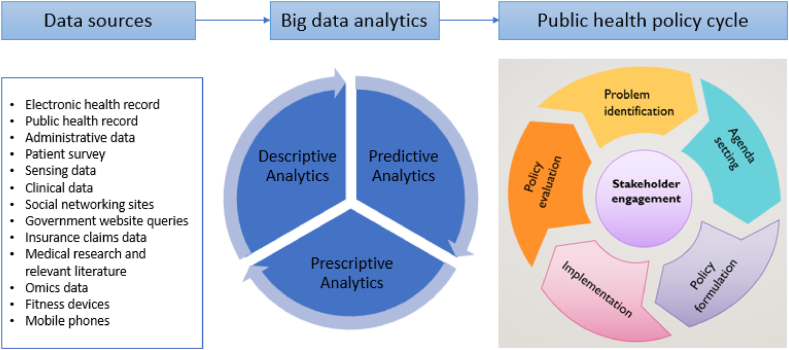
BDA-driven public health policy framework.

A graphical model has been used to present the conclusion (Fig. 4). The model consists of four basic phases, including the primary sources of big data, a processing unit, the creation of data-driven public policies, and the implementation of such policies. Public census data, in-person interviews, crowdsourced data, private sector transactions, social networking sites, website requests from the government, public development sector, tax information, and routine inquiries from various stakeholders to public agencies are the main sources of big data. The processing unit primarily handles the data warehouse and different features. The process of creating public policy involves four phases: public monitoring, public regulation, public service delivery and policy feedback.

### 4.1 Data sources and public health policy

Gathering and analyzing substantial volumes of data from several sources is considered big data analytics in the healthcare and medical fields. These sources can include, among others, mobile health [86], social media platforms [61,68], medical literature [79,83], physician notes, prescriptions, medical imaging, laboratory reports [59,66], biomedical research [73], omics data [88], and machine- and sensor-generated data [57,64]. Huge data sets generated by the technologies mentioned above are being developed concurrently with new techniques for acquiring, storing and processing data to improve its availability and usability [54,61]. Additionally, new logical and analytical methods are continually being created. Due to improvements in information technology, both in terms of hardware and software, the ability to analyze and interpret data has reached new heights [89]. Information may now be accessed and used in previously impractical ways, thanks to the digitization of data and related data flexibility, advancements in artificial intelligence and computer thinking, computerization, automation of various processes and increases in computing power. The public and business sectors are becoming increasingly aware of the availability of data and creative uses for it [75,82]. Many government agencies have put big data plans or policies into place [84,90].

The growth of big data has had a variety of advantageous consequences for several businesses. One area that might profit from big data insights to improve public service satisfaction is the public sector [[91], [92]]. A big data deployment for a company has benefits, such as social, historical, and predictive analysis. BDA improves decision-making accuracy and boosts data variety through objective assessments, which will affect the considerations advanced during policy planning. Big data technologies are frequently used in the public sector to collect responses from the general public, which are then used to improve public services and shape policy.

### 4.2 Analytics for public health policy

The BDA tools can transform public health policy in various ways. While governments are acclimating to the most recent tools and technology more slowly than the private sector, the latter is quickly adopting data techniques for decision-making [94]. Many public agencies are not ready to use big data analytics for their strategic advantage, which entails optimizing workflows, redefining roles and actions, taking into account the disruptive nature of technological advancements, and researching the subject broadly to find advantages for policy development, definition and evaluation [94]. Technology is still seen as an additional tool to the current organizational and administrative procedures for governmental acts, but scarcely seen as game changer to the underlying components of decision-making processes. Privacy and data security concerns often hinder participants, suppliers, and facilities from sharing data, even within the same organization. This is a primary reason for the slow adoption of big data in healthcare. Making effective risk estimates and evaluations, meaningful resource allocations, and foreseeing potential unintended effects are all goals of public health policy-making [[95], [96]]. Therefore, relying on pertinent and timely research to promote participatory interventions is crucial for effective public health policy-making [71].

Big data technology makes it possible to swiftly and non-persistently match and connect together pieces of related but heterogeneous information to detect as-yet-undiscovered information flows. Hidden connections and patterns will be found to back up common sense or conventional knowledge. Applying predictive analytics on top will improve scenario planning and produce genuine evidence-based policymaking. Organizations will learn about how they operate and how their clients or citizens utilize them [97], and will build services appropriately, since changes to the organizational structure are necessary to exploit the potential benefits of BDA. BDA can easily identify the areas of underperformance. The ability to examine many data sources and identify trends makes this possible. As a result, less time will be needed to prepare reports, which means more time can be spent on complex analyses [11].

The BDA-improved procedures will minimize paperwork for the residents, as internal process reorganizations that ensure better data integration for analytics will allow collaboration among ICT systems, which decreases the need for individuals to supply the same information continually. Consequently, citizens will obtain their benefits, and questions will be handled more swiftly. Large-scale predictive analytics may also result in services being proactively suggested based on those utilized by similar citizens.

### 4.3 BDA for public health policy cycle

#### 4.3.1 Problem identification

Identifying and clarifying the problems that could be the focus of public health policy, is concerned with how issues are identified as requiring government attention. Data and information must support evidence, and the more reliable the information that is accessible throughout a decision-making process, the greater the quality of the conclusions will be [22]. However, improving attribute information alone as opposed to overall information quality would lower decision quality [98]. We observe an increasing unwillingness to implement filters to decrease a problem's complexity, since very little data is ever eliminated. Big data advocates assert that data classified as noise contains potentially important information, but sophisticated approaches and algorithms for separating this important data from noise are not commonly used [99]. Given that connections may be discovered automatically via machine learning algorithms, data can be examined as its whole, and analytical findings could become available instantly. Big data, especially BDA, promises to provide quicker and better insights. One comparative benefit of applied BDA that we will discuss in connection to the phases of the policy cycle process is the capacity to respond promptly to unfavorable repercussions of a decision.

#### 4.3.2 Agenda-setting

Many researchers claim that the issue definition and agenda-setting steps of the policy process involve the usage of big data [31,40,57]. Big data may be used as a tool to “define a policy problem before it is seen as such, revealing where demand is being satisfied or where an emerging problem may be countered early.” The media play a critical role in setting agendas by framing subjects and delivering relevant information. Digital media further exacerbate agenda-setting dynamics. Social media allows for speedy introduction of fresh topics by any audience member, and participation in ongoing discussions can take many forms, including text, voice, video and graphics [68,93].

#### 4.3.3 Policy formulation

A policy is created by outlining the actions that must be performed during the implementation stage. The two phases of policy development and acceptability have distinct relationships with (Big) data because they are deeply rooted in the legal code governing how governments must behave. However, big data has a place in the field of appraisal. Once the policy has moved from the deliberation phase to the policy formulation phase, the policy papers may be examined and governments can approve or create actual policies in response to public demands [66]. It will be a beneficial endeavor to employ means of data collection to explore the adoption of particular policies among different sectors of society, since the credibility and legitimacy of new policies, especially in democracies, are vital. In the digital age, the concept of acceptance may be extended to include public acceptance as well as the political act of voting by elected officials. Using sophisticated predictive analytics approaches and scenario tactics, BDA may help evidence-based policy making during the policy creation and adoption cycle stages [63].

The idea of policy design is related to the notion that governments work to achieve goals effectively and efficiently and are interested in leveraging information and skills relating policy difficulties [87]. After a public problem has been put to their official agenda, government authorities can create comprehensive action plans [83]. Policy formulation includes developing various action plans for addressing (resolving or ameliorating) societal issues. Most design work is done at the formulation stage. This perspective gives policy instruments a lot of thought. The decision-makers think about both what to do and how to do it while evaluating various policy possibilities. Giest [83] points out that the expanding use of big data is impacting the design of policy instruments by connecting these concepts to big data. Due to digitalization, the vast administrative data can be collected at many levels of government and in a variety of areas, and may be utilized to guide choices in the areas of education, economics, finance, health and social policy. The different ways that big data may be used to further particular policy objectives are illustrated by the variety of information-based policy tools used by governments. Procedural informational devices depict government efforts to regulate information. By controlling and distributing information in a way that is compatible with the goals and objectives of the government, they hope to have an impact on the policy-making process.

While some programs aim to limit information availability, others want to stimulate it. Two examples of how big data may be used as procedural policy instruments are government data releases and open data policy frameworks [73]. Government collect data from important informational sources including judicial investigations, executive commissions, the national statistics agency, surveys and polls to support evidence-based policymaking. Governments are now increasingly fusing big data based on input from social media, cameras and sensors with these more traditional statistics [83]. Giest [83] and Williamson [69] assert that these techniques provide policymakers with up-to-date information on the educational system by forming interactive and digital data visualizations.

#### 4.3.4 Policy implementation

Big data has the potential to affect policy implementation in two different ways: The first possibility is that varying levels of policy intensity might be implemented by being able to identify problem areas. For instance, by increasing enforcement, problem areas may be targeted with greater specificity, thereby reducing crime before it starts. Second, the adoption of new policies will almost instantly result in the generation of new data that can be used to assess their efficacy and improve subsequent implementation procedures by identifying issues with the current ones. As will be shown in relation to the assessment stage, the new evaluation dimension in particular will probably have the biggest impact on the various policy cycle phases. When it comes to transforming policy ideas into truly executable policies, the generation of data during—not after—the execution of policies offers unprecedented flexibility [95]. For instance, a new redistributive tax law might be evaluated almost immediately to see if it has the desired impact or if adjustments are required.

Big data can improve the accuracy of some of the key information sources used to implement policies. When utilized to develop and put into effect new laws, census data, for instance, frequently runs the danger of being out of date. However, instead of being updated once or twice every ten years, census data might be created on a nearly daily, rolling basis through the integration of many databases. Real-time monitoring of demographic information, unemployment rates or migration trends enable to the rapid assessment of the success or failure of a particular policy's implementation [75]. The addition of external data would be a good next step, either to improve the validity of the data cross-check process or to augment the existing authoritative government data [59].

#### 4.3.5 Policy evaluation

Policy evaluation step evaluates how a public policy has performed in practice, including its outcomes, efficacy and justifications [100]. It requires an evaluation of the methods administered and the aims being sought [75]. A policy may be revised after evaluation, or the status quo may be upheld. Big Data's analytic capabilities apply to the assessment element of policy cycle [101]. Evaluation occurs at the end of the policy-making process in the typical policy cycle, although early exits from earlier process phases are made as soon as the failure is clear. However, in this case, changing the predetermined agenda is problematic, since, prior to the advent of Big Data, typical health policy systems' assessment delivery times were frequently insufficient to support early departures from the policy cycle. The ability to handle data in real-time is one of the Big Data toolbox's defining features. Instantaneous or almost instantaneous data processing can make evaluation findings available as soon as new data is received. A new perspective on the policy cycle is therefore made possible, namely the idea of continual evaluation. With the use of BDA, evaluation may take place at any point and in a way that is secret to the parties involved in the policy cycle.

### 4.4 Challenges of implementation of data-driven policy

Government organizations must understand data security and privacy. Some governments have open data policies that might lead to a catastrophe if terrorists or powerful interests use the data to their advantage or the benefit of other countries [89]. This security issue needs to be properly watched by the government organization. Since citizen data is used to make choices, capture criminals, diminish corruption and advance social welfare, the government must preserve its citizens' privacy [98]. This underscores the need for the government to create a safe system before implementing big data technologies.

Government agencies use big data analytics to make swift judgments. However, the require data must be error-free, comprehensive and supplied on schedule for immediate usage. Some government entities use standardized formats and metadata to maintain data accessibility and flow [102]. Open data policies are prevalent today, making data sets available to the broader public and facilitating collaboration across several agencies. However, these policies must be done in accordance with privacy laws [103]. For smart governance, there must be a constant flow of reliable data that is accessible, discoverable and useable [89].

Low-cost memory, storage, cloud-based solutions, and highly effective servers and platforms are required for big data technology [97,98]. One of the finest technologies for leveraging big data in the public sector is cloud computing. It is simple to employ for flexible computational studies by government agencies. Government organizations should make sure there is enough bandwidth and real-time data analysis when employing a cloud environment so they can make quick, educated decisions [104]. Government agencies should collaborate with other internal and external entities to transform data and reduce technical challenges [105]. In order to aid the government in all areas and lessen risk and hazard to government agencies, a team of professionals is required to collect, handle, process and manage a significant quantity of data. This is crucial for putting into place and maintaining smart governance structures.

Big data could lead to exaggerated claims of objectivity and accuracy. There is a considerable gap in science between qualitative and quantitative scientists. It would seem that while quantitative scientists would be in charge of producing facts, qualitative scientists would be involved in conveying and understanding stories. That is untrue since everyone who makes an objectivity claim also makes subjective observations and judgments. In addition, many assumptions are made when processing data, and the interpretation of results is subjective. Reliably integrating disparate datasets and the possible effects of frictions and self-selection on internet databases are additional problems. This viewpoint asserts that big data may support claims of objectivity and accuracy that are not corroborated by reality or common sense [74].

Data volume and quality are not always connected. Every field of study has a substantial body of literature that strives to guarantee the uniformity of data collection and interpretation [19]. Big data scientists assume that their data is of a certain quality rather than considering the methodological issues related to data quality. The level of the scientific evidence must thus be regulated and the underlying assumptions of the research findings must be made clear, even though big data allow managers and decision-makers to base judgments on evidence rather than just gut feeling [87]. Data fusion does not come without a warning. It is necessary to adhere to any privacy and data protection laws already in place [89]. Big data's potential to undermine privacy and other values and its socially beneficial uses are delicately balanced [106]. This brings up complex issues on how to make sure that discriminatory impacts brought on, say, by automated decision-making systems, may be found, assessed and corrected. Having thorough understanding of the populace enables highly accurate public behavior predictions [107]. A system of checks and balances and responsible leadership are necessary for this authority. There is no longer any justification for ignoring tradeoff considerations due to the increased possibility of a significant loss of informational privacy-related benefits [6]. The government must advance this agenda with high ethical standards since big data has great promise but also has the potential to restrict human liberties.

It is also necessary to consider the environmental impact of BDA operations. Large-scale dataset processing, analysis, and storage require massive computing power, which impacts energy use. The development of BDA technologies has made it imperative to consider these environmental effects and devise mitigation strategies [108]. Policymakers should consider these issues during health policy decisions. Besides, the reliability of AI in health policy formation should be ensured as it impacts enormous populations. Reliability, openness, and correctness of analysis powered by AI must be guaranteed. Ho et al. [109] argued that relevant stakeholders must consider a human-centered approach to the design and use of AI and BDA, and legislative guidelines must be implemented.

### 4.5 Recommendations to address challenges in health policy decision

Healthcare and governance are changing due to the use of BDA in formulating public health policies. Big data offers benefits, but there are also significant obstacles. This study recommends several strategies to address major challenges.

For data security and confidentiality, innovative encryption strategies and the creation of privacy-preserving analytics tools can be used to draw insightful conclusions without disclosing specific data [110]. Putting multi-factor authentication, secure data transfer methods, and sophisticated encryption into practice can protect against data breaches [2]. Accurate, thorough, and timely data are necessary for quick and efficient decision-making. Standardized metadata and data formats should be used together with strong validation techniques to ensure data reliability. Tools for real-time data collection and analysis may also aid in timely utilization.

Cost-effective devices, tools, and storage solutions are also needed. Cloud computing can be an effective solution to handle large amounts of data [111]. There must be sufficient bandwidth and real-time analytic capabilities to ensure prompt and well-informed decision-making. Implementing redundancy, failover plans, and high availability should provide continuous data services.

Training programs and awareness initiatives may assist stakeholders in identifying and reducing any biases [2]. Quantitative, and qualitative perspectives should be focused on achieving balance and effective outcomes. Collaboration between quantitative and qualitative researchers, transparent algorithms, and interpretability tools may all help to promote balanced knowledge [112]. To achieve high-quality data-based results, regular audits, validation tests, and data quality frameworks might be helpful.

## 5 Conclusions

This research gives readers a conceptual and practical understanding of the possibilities of big data in the public health policymaking process. It also offers a wide viewpoint on big data trends in the public health literature to pinpoint potential areas of future research and offer examples from public health policy domain. In the planning stage, the potential of big data has been highlighted in the aspects of agenda-setting, problem-defining, policy-discussing and citizen involvement. The creation of information-based policy instruments and the formulation of policies during the design phase may be aided by BDA. Many of the techniques being employed at this point include a predictive element. The delivery phase concentrates on immediate response concerning the effectiveness of policies and data production in real-time to enhance future implementation processes. Throughout all phases of the policy-making process, BDA may also be utilized to review policies continually.

This study argues that BDA is applicable to the policy identification, agenda setting, policy formulation, policy implementation and policy evaluation of a health policy cycle. Descriptive, predictive and prescriptive analytics are used for public health policy decisions on the data obtained from common health-related big data sources like electronic health reports, public health records, patient and clinical data, and government and social networking sites. The major health policy stakeholders commonly face several challenges while handling health-related big data, such as privacy and security, data sharing challenges, technology-related challenges, skill-related challenges, data modeling, data management issues, and data services and tools.

The basic ideas underlying big data are neither fundamentally novel nor revolutionary; rather, we observe a repackaging of terms that later enter the big data arena. Making choices in real-time is novel, and it has the potential to displace the conventional model of the policy cycle's sequential execution with a continuous assessment model. Obtaining more valuable information from data otherwise categorized as noise can significantly accelerate decision-making and result in better conclusions. BDA approaches also make it simpler to involve the general public at different stages of the cycle since it is simpler to handle the enormous volumes of unstructured data collected to account for the crowd's wisdom. We have demonstrated how BDA may support the health policy cycle in some ways. However, iteratively analyzing the policy cycle is where the largest gains may be obtained, particularly when employing big data-enabled approach.

Instead of focusing on a single application, our contribution to the field of policy analysis is to advocate the use of BDA at each step of the health policy-making process, from formulation to evaluation. This is made possible by technological advancements that enable access to, processing, analysis, and storage of enormous volumes of diverse data. However, one must be careful not to become unduly fascinated by technological advancements. These innovations, nonetheless, occur in a bureaucratic environment, complete with its peculiarities and organizational cultures, where each participant is presumably determined to take benefit of the application of new technology. Since different government traditions and cultures are related to the adoption of technologies in different ways, determining the right level of technical capability, openness and autonomy for government in the digital era is still a challenging task for which there is no comprehensive solution. Another more common challenge to adopting BDA-enabled evidence-based policymaking is the involvement of politicians, who embrace evidence when it supports their position and reject it when it does not. An effective countermeasure would be to organize the information so that anybody interested could go further into the data, enabling the general public to form opinions based on easily available facts and figures. Even the most stubborn politicians are powerless to deny the facts' indisputable veracity.

Several different groups, including public health officials, members of the healthcare sector, specialists in technology and data science specialists, patients, and the general public, can benefit from the study's findings. For example, policymakers will gain from the results by learning how to use BDA for successful and evidence-based policy-making. Medical practitioners may use the findings to enhance patient outcomes and service delivery. Technology and data science professionals will determine the route for creating more effective, dependable, and useable BDA solutions for the healthcare industry. Implementing better-informed, more efficient public health policies that might raise the standard of treatment and improve health outcomes would benefit patients and the broader population.

## 6 Limitations of the study

Our article has several limitations. First, we lack primary data; therefore, we depend on carefully chosen and well acknowledged scientific literature to draw our findings. Future studies can be conducted based on primary data collected from relevant industries to present the real impact of BDA on health policy formulation and implementation. Second, this study mainly used the Scopus database for the systematic literature review. Future review studies should take more databases, such as the Web of Science, PubMed, and Engineering Village, for in-depth research. Third, although enticing, the reality of continuous evaluation has not yet been tested. Future studies can develop a prototype of a continuous evaluation system using real-world data to show the real-time effectiveness of health policies. Finally, we recognize the importance of introducing the BDA early in the public health policy process to get a maximum outcome. Future studies can address this issue to determine its applicability in the context of a health administrative system.

## 7 Research gap and future research direction

Public health policies, including laws, regulations and standards significantly impact public health. However, there is a large discrepancy between the policies that are implemented and enforced and what research considered beneficial. The most probable way that research will affect how policies are developed is through a prolonged communication and engagement process. Pseudo-evaluation, formal assessment and decision theoretic assessment are just a few of the tools and approaches that analyst might use to help them evaluate health policy. Due to the interdependence and variety of both programming languages and approaches, their integration into a practical framework for policy making is still far from being accomplished. Insights from data obtained from research, clinical care settings and operational settings may be captured using big data analytics to create evidence for better care delivery.

To make BDA consumption more sustainable, research should be done on the energy efficiency of data processing and AI model training. The openness, interpretability, and fairness of AI systems employed in policy formulation and their trustworthiness are crucial research topics. It also necessitates paying careful attention to ensure the ethical use of data and privacy protection. A further way to guarantee the overall sustainability of AI-assisted policies is to evaluate their social, economic, and environmental effects.

The speed at which future policy choices are made will be accelerated, expenses will be decreased, and effectiveness will be raised by adding big data-enabled updated policy cycle. As the administration broadens continuous evaluation across all governmental levels and businesses, employee and contractor privacy should be carefully reviewed. The process for clearing data must give participants the chance to correct or challenge inaccurate data that led to the production of assessment findings. BDA should be used as an instrument in the public health policy system that helps a lot of people. If a decision-maker is hesitant to implement a BDA-based plan, the related endeavor should not be approved as a government policy.

The political actors' commitment will still play a significant role in the true transformation of health policymaking processes and the transition from estimate-based to evidence-based policies. Many nations are presently making an effort to set up independent auditing organizations to assess the effectiveness of initiatives. For these advancements to be successful, it is essential to consider the current state-of-the-art in ICT and BDA. Pre-existing frameworks should not constrain BDA-based models of governance and policymaking for decision-making if the health sector keeps up with the pace of the growing needs of the citizenry. BDA should be viewed as an artefact, and the focus should move from implicit theorizing about generalized technologies to explicit theorizing about individual technologies.

## Funding

This research received no external funding.

## Data availability

The data will be available on request.

## Author contributions

Conceived and designed the experiments: Kang Chao, and Md Nazirul Islam Sarker. Performed the experiments: Kang Chao, Md Nazirul Islam Sarker, Isahaque Ali, and R. B. Radin Firdaus. Analyzed and interpreted the data: Kang Chao, Md Nazirul Islam Sarker, Isahaque Ali, R. B. Radin Firdaus, Azlinda Azman, and Maslina Mohammed Shaed. Contributed reagents, materials, analysis tools or data: Kang Chao, Md Nazirul Islam Sarker, Isahaque Ali, R. B. Radin Firdaus, Azlinda Azman, and Maslina Mohammed Shaed. Wrote the paper: Kang Chao, Md Nazirul Islam Sarker, Isahaque Ali, R. B. Radin Firdaus, Azlinda Azman, and Maslina Mohammed Shaed.

## Declaration of competing interest

The authors declare that they have no known competing financial interests or personal relationships that could have appeared to influence the work reported in this paper.
